# Unsupervised Analysis of Classical Biomedical Markers: Robustness and Medical Relevance of Patient Clustering Using Bioinformatics Tools

**DOI:** 10.1371/journal.pone.0029578

**Published:** 2012-03-05

**Authors:** Michal Markovich Gordon, Asher M. Moser, Eitan Rubin

**Affiliations:** 1 Sharaga Segal Dept. of Microbiology and Immunology, Ben Gurion University of the Negev, Beersheba, Israel; 2 The National Institute for Biotechnology in the Negev, Beersheba, Israel; 3 Dept. of Pediatric Hematology and Oncology, Soroka Medical Center, Beersheba, Israel; 4 Department of Pediatric Hemato-Oncology, Ben Gurion University of the Negev, Beersheba, Israel; Genentech Inc., United States of America

## Abstract

**Motivation:**

It has been proposed that clustering clinical markers, such as blood test results, can be used to stratify patients. However, the robustness of clusters formed with this approach to data pre-processing and clustering algorithm choices has not been evaluated, nor has clustering reproducibility. Here, we made use of the NHANES survey to compare clusters generated with various combinations of pre-processing and clustering algorithms, and tested their reproducibility in two separate samples.

**Method:**

Values of 44 biomarkers and 19 health/life style traits were extracted from the National Health and Nutrition Examination Survey (NHANES). The 1999–2002 survey was used for training, while data from the 2003–2006 survey was tested as a validation set. Twelve combinations of pre-processing and clustering algorithms were applied to the training set. The quality of the resulting clusters was evaluated both by considering their properties and by comparative enrichment analysis. Cluster assignments were projected to the validation set (using an artificial neural network) and enrichment in health/life style traits in the resulting clusters was compared to the clusters generated from the original training set.

**Results:**

The clusters obtained with different pre-processing and clustering combinations differed both in terms of cluster quality measures and in terms of reproducibility of enrichment with health/life style properties. Z-score normalization, for example, dramatically improved cluster quality and enrichments, as compared to unprocessed data, regardless of the clustering algorithm used. Clustering diabetes patients revealed a group of patients enriched with retinopathies. This could indicate that routine laboratory tests can be used to detect patients suffering from complications of diabetes, although other explanations for this observation should also be considered.

**Conclusions:**

Clustering according to classical clinical biomarkers is a robust process, which may help in patient stratification. However, optimization of the pre-processing and clustering process may be still required.

## Introduction

Advances in medical informatics, in bioinformatics and in machine-learning are opening up new venues in biomedical research. Data is becoming ever more accessible as information from large populations of patients is now electronically captured, especially in countries with centralized healthcare. A general move toward lifelong Electronic Health Records (EHRs), aimed at integrating available clinical information for an individual, has been suggested [1], [2]. In parallel, the computational tools required for analyzing complex, biased and error-ridden datasets are constantly improving.

One possible role for computational analysis of clinical data lays in improving our ability to understand disease sub-classes, or stratification. Medical research, as required for use by treating physicians, often attempts to define disease sub-classes that are more homogenous in terms of optimal intervention and prognosis. More refined classifications can, however, help physicians in achieving ‘personalized medicine’, in which intervention is tailored to specific patients [3], [4]. While great emphasis is currently being given to identifying biomarkers that will allow adequate stratification of patients, it has been shown [5], [6] that existing clinical data, when analyzed with unsupervised learning methods (i.e. clustering), can be used to uncover classification patterns. According to this approach, patients are grouped based on a combination of clinical observations (e.g., laboratory test results, symptoms, complaints, etc.) and the resulting clusters are tested for enrichment with patients with similar outcomes or responses to a given treatment. Claycamp *et al.* (2001) [6] grouped patients suspected of having chronic radiation sickness based on blood count values. Using competitive artificial neural network clustering, they found a cluster enriched with true chronic radiation sickness patients. Chen *et al.* extended this idea to develop a ‘clinarray’, in which the entire spectrum of clinical laboratory tests results was used to cluster children with similar profiles. Using agglomerative hierarchical clustering, these authors were able to sub-classify Crohn's disease and cystic fibrosis patients in a manner that correlated with the severity of their disease [5].

While these works demonstrate the potential of utilizing machine-learning methods commonly used in bioinformatics (namely, clustering), little is known about the robustness of this approach. In both studies mentioned, the ability to classify patients was not repeated with additional datasets, and the sensitivity of the process to methodological decisions was not assessed.

In this study, we demonstrate the power of unsupervised learning in clinical data analysis, while evaluating the robustness of this approach. To achieve these goals, we designed a training-validation experiment in which the quality of the clusters resulting from biomarker-based clustering are evaluated in a second dataset. Two large datasets derived from the National Health and Nutrition Environmental Study (NHANES; NHANES website. Available: http://www.cdc.gov/nchs/nhanes.htm Accessed 2011 Dec 4.) were used. The NHANES is a prospective cross-sectional study in which health state, lifestyle parameters, and a comprehensive set of laboratory tests have been recorded for thousands of random, non-hospitalized USA residents. The quality of clusters generated with various pre-processing and clustering methods was compared to evaluate the robustness of a given clustering approach to methodological decisions. In addition, the robustness of the clusters to sample choice was evaluated by projecting the resulting clustering scheme from a training to a validation dataset, and comparing the enrichment of the resulting clusters from both datasets with clinical and lifestyle properties. Our results indicate that the methodology chosen affects the quality of the resulting clusters, and suggest that clusters are indeed robust across datasets. We further demonstrate the potential value of clustering to disease stratification, showing that clustering diabetic patient by their classical biomarker values reveals biomedically interesting and non-trivial sub-classes.

## Results

**Figure 1:**
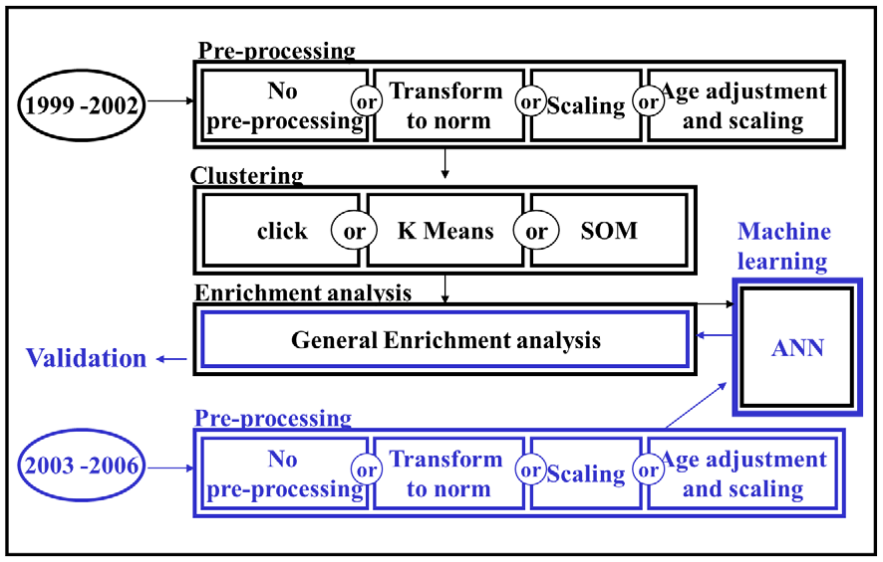
Methodology overview. A test-validation approach was used to test the impact of methodological choices on the clustering of individuals according to their classical blood biomedical marker values. The data from the NHANES 1999–2002 surveys was used as a training set, while the 2003–2006 dataset was used for validation. Various combinations of pre-processing and clustering algorithms were used to define clusters from the training set (black). For pre-processing (top row), transformation to normal of otherwise non-normal variables, Z-score normalized and Z-score normalized-with age adjustment using linear regression, were considered (top block). Each resulting dataset was clustered with three different clustering algorithms (second row): CLICK [18], K-means [21] and self-organizing maps [22]. The resulting clusters were used for enrichment analysis with health/lifestyle traits and for training an artificial neural network (third row). The artificial neural network was subsequently used to assign individuals from the validation set to clusters (third row), using the same pre-processing procedure as used to generate the training set clusters (bottom row). The resulting validation set clusters were also tested for enrichment with the same health/life-style traits as the training set. Enrichments found in both sets were compared.

To evaluate the robustness of patient clustering by classical marker values, we derived two datasets (i.e. training and validation) from the NHANES Study, each comprising data collected in different years from a random sample of non-hospitalized individuals. The training dataset was processed with 12 different analysis pipelines (Figure 1), each involving one of four pre-processing methods and one of three clustering algorithms. For each pipeline, an artificial neural network (ANN) was used to cluster the validation set into the same clusters as defined based on the training set. Enrichment analysis with a set of known diseases and complaints was then performed on the clusters both in the training and validation sets, and their consistency was compared.

### 2.1 The impact of different pre-processing and clustering algorithms on the properties of the resulting clusters

Initial comparison of the clusters obtained with the different clustering pipelines was performed using the H_A_-S_A_ measure. This measure reflects the difference in variation between individuals in different clusters (S_A_) and individuals in the same cluster (H_A_), with high values indicating well defined clusters (i.e. distant and compact) and low values indicating poorly separated clusters.

Only small differences were observed in the homogeneity of the clusters obtained with the three different clustering algorithms (Table 1), when the same pre-processing approach was used. Clusters generated with Z-score-normalized data, for example, had mean H_A_-S_A_ values of 0.554±0.02, 0.644±0.156 and 0.467±0.06 with the CLICK, K-means and SOM algorithms, respectively. All of these differences were found to be statistically significant by Student's t-test, using p<0.005. A much larger impact was observed when different pre-processing methods were used. Z-score normalization dramatically improved the quality of the clusters. For example, with the CLICK algorithm, a 10-fold increase was observed in the mean H_A_-S_A_ value between scaled and unprocessed data, growing from 0.035±0.005 to 0.554±0.02 (p<0.001, Student's t-test; see Table 1).

**Table 1:** The quality of clusters obtained with different pre-processing-clustering pipelines.

	Processing pipeline		Training		Validation	
			N	H_A_-S_A_	N	H_A_-S_A_
**All Patients**	CLICK	Raw	7	0.035 (0.005)	6	0.05
		NormTransf.	8±1	0.012 (0.003)	6	0.013
		Z-Score	24±3	0.554 (0.02)	24	0.556
		AgeAdj	21±3	0.54 (0.02)	17	0.532
	K-mns	Raw	7	0.022 (0.036)	7	0.031
		NormTransf.	8±1	0.01 (0.0036)	6	0.011
		Z-Score	24±3	0.644 (0.156)	18	0.522
		AgeAdj	21±3	0.647 (0.174)	14	0.534
	SOM	Raw	8	0.026 (0.016)	7	0.032
		NormTransf.	8±1	0.009 (0.003)	6	0.008
		Z-Score	20±30	0.467 (0.06)	19	0.451
		AgeAdj	20±3	0.44 (0.076)	17	0.43
**Diabetes**	CLICK	Raw	0	-	-	-
		NormTransf.	3	0.033 (0.001)	4	0.033
		Z-Score	5±1	0.64 (0.02)	6	0.622
		AgeAdj	5±1	0.72 (0.04)	6	0.619

For each pre-processing/clustering pipeline, the number of clusters (*N*) and the homogeneity-separation difference (*H_A_*-*S_A_*) are provided. The *H_A_*-*S_A_* measure reflects the difference in variation between individuals in different clusters (*S_A_*) and individuals in the same cluster (H_A_). For the validation set, the mean±SD of the number of clusters and *H_A_*-*S_A_* were calculated for 10 re-sampled datasets. See Materials and Methods for a complete description of the algorithms and pre-processing methods. *K-mns* = *K-means*; *NoramTranf* = transformation to normal; *Z-score* = Z-score normalization; *AgeAdj* = age adjustment followed by Z-score normalization. The analysis was performed separately for the NHANES training and validation sets, using all individuals (“All Patients”) or restricting the analysis to diabetic patients (“Diabetes”).

The effect of age adjustment for this dataset is not as clear. On the one hand, no striking improvement was observed in cluster quality, as reflected in their highly similar H_A_-S_A_ values with or without age correction (e.g. 0.554±0.02 vs. 0.540±0.02 using Z-score normalization with or without age correction, correspondingly, applying the CLICK algorithm in both cases). On the other hand, the number of clusters identified using the CLICK algorithm with age-adjusted before-Z-score data was smaller than the number of clusters identified with Z-score-normalized data with equivalent H_A_-S_A_ values. This could reflect some age-based clustering in the non-adjusted data, where individuals that otherwise very similar are assigned into different clusters based on age alone. These results thus suggest that age normalization may have improved the quality of the clusters identified using the CLICK algorithm, as it generates fewer clusters without compromising cluster homogeneity.

### 2.2 The robustness of biomarker-based clustering across datasets

To test whether clusters identified via unsupervised analysis are robust across datasets, we projected the clusters generated with the test set to the validation set using an ANN, and compared the enrichment observed in the clusters from either set in terms of health/lifestyle properties. We assumed that if the clusters resulting from grouping individuals based on their biomarker values are robust to dataset effects, then the resulting clusters should be similar in terms of their enrichment patterns. To determine whether this is indeed the case, we first tested whether the clusters generated from the training dataset can be successfully identified in the validation set. For most clusters, a cluster with similar quality could be detected in the validation dataset, as reflected by their H_A_-S_A_ values (Table 1). For the CLICK algorithm, for example, all 24 clusters generated from the training data were successfully recovered in the validation set. We then tested whether the clusters produced from the validation dataset had the same biomedical meaning as did the training set clusters by comparing their enrichment with health/lifestyle properties of the individuals included. Nineteen health/lifestyle-related statements were extracted from the NHANES datasets, choosing traits pertinent to a sufficiently large subset (n≥30; see Table S1b for complete details). These include mostly diseases (e.g., diabetes, coronary heart disease, etc.) but also some lifestyle traits (e.g., smoking). Three measures were used to evaluate the robustness of clinical biomarker clustering: (i) The number of *valid enrichments*, defined as significant enrichments of the same trait in equivalent clusters from both training and validation datasets, (ii) the number of *distinct validated enrichments*, defined as the number of distinct terms that were successfully validated and (iii) *validated clusters*, defined as the number of clusters in the validation set with one or more validated enrichments (Table 2). A complete list of all validated enrichments is provided in Table S3.

**Table 2:** Comparative enrichment analysis of biomarker-based patient clusters: A quantitative analysis.

Processing pipeline			Valid enrichments		Validated clusters	Enrichment factor
			Total	Distinct		
**All Patients**	CLICK	Raw	7/12	4/9	5/5	4.3
		NormTransf.	12/16	8/12	4/5	4.3
		Z-Score	22/40	15/20	8/13	5.9
		AgeAdj	20/38	11/20	8/9	5.3
	K-mns	Raw	7/11	5/6	5/6	4.5
		NormTransf.	11/17	9/14	5/5	4.2
		Z-Score	18/37	11/16	7/17	6.4
		AgeAdj	11/34	7/15	7/15	7.0
	SOM	Raw	8/20	4/12	5/7	2.9
		NormTransf.	11/13	7/9	5/6	1.9
		Z-Score	36/62	13/21	8/16	7.7
		AgeAdj	14/28	8/15	8/10	3.8
**Diabetes**	CLICK	Raw	0/0	0/0	-	-
		NormTransf.	0/2	0/2	0	-
		Z-Score	2/4	2/3	1/2	-
		AgeAdj	4/7	4/6	1/2	1.8

The number of validated terms found to be enriched in clusters generated with the different pre-processing procedures and clustering algorithms tested in this study. Validated enrichments and validated clusters are defined by the recurrence of statistically significant enrichment in the training and validation datasets. The clusters that were generated from the test dataset using a particular pre-processing clustering combination were subjected to enrichment analysis with 19 health/lifestyle labels (i.e. searching for statistically significant over-representation of patients with the trait in each cluster). An artificial neural network, trained with the cluster assignment of each individual in the training dataset, was used to classify individuals from the validation dataset using the same clinical biomarkers subjected to the same pre-processing algorithm as was the test dataset. The resulting clustering of the validation set was also subjected to enrichment analysis with the same terms as was the training set. An enrichment was deemed to be a validated enrichment if the same label was enriched in the test and validation datasets. A validated cluster was defined as a cluster sharing at least one enriched term between the test and validation sets (i.e. the number of clusters enriched in the training set). The enrichment factor for each pipeline is the average enrichment factor of the three most significant enrichments. *K-mns* = *K-means*; *NoramTranf* = transformation to normal; *Z-score* = Z-score normalization; *AgeAdj* = age adjustment followed by Z-score normalization.

Analysis of the enrichment results suggest that use of the SOM algorithm with Z-score normalization gives the most specific enrichments. This pipeline produced eight validated clusters (as compared to four-eight generated by the other pipelines) and an average fold-enrichment of 7.7 (as compared to 1.9–7 for the other pipelines). The SOM/Z-score normalization algorithm also had the highest absolute number of validated enrichments (36, as compared to 8–33 for the other pipelines). However, this algorithm did not generate the highest number (or fraction) of distinct validated enrichments. The SOM/Z-score normalization algorithm yielded 13 distinct validated enrichments, as compared to 15 using the CLICK algorithm with the Z-score normalization pipeline. In fact, detailed analysis of the enrichment obtained with the different algorithms using scaled data reveals that the CLICK algorithm finds all the enriched validated terms that are found by the two other algorithms (Table 3A). In terms of pre-processing, scaled data yielded the highest number of validated enriched terms when using the CLICK algorithm (15 compared to 11). However, the use of age-adjusted pre-processing added one term (“taking treatment for anemia/past 3 month”) that was not found with the scaled data (Table 3B).

**Table 3:** Enrichment analysis of biomarker-based patient clusters: A qualitative view.

	Scale					
	Code	Enrichment name	CLICK	Kmns	SOM	
**Z-score normalization**	DIQ010	Doctor told you have diabetes	Y	Y	Y	
	KIQ020	Ever told you had weak/failing kidneys	Y	Y	Y	
	MCQ160A	Doctor ever said you had arthritis	Y	Y	Y	
	MCQ160B	Ever told had congestive heart failure	Y	Y	Y	
	MCQ160C	Ever told you had coronary heart disease	Y	Y	Y	
	MCQ160E	Ever told you had heart attack	Y	Y	Y	
	MCQ160F	Ever told you had a stroke	Y	Y	Y	
	MCQ160G	Ever told you had emphysema	Y	Y	Y	
	MCQ190	Which type of arthritis	Y	Y	Y	
	MCQ220	Ever told you had cancer or malignancy	Y	Y	Y	
	MCQ160D	Ever told you had angina/angina pectoris	Y		Y	
	MCQ140	Trouble seeing even with glass/contacts	Y		Y	
	MCQ160L	Ever told you had any liver condition	Y		Y	
	SMQ040	Do you now smoke cigarettes	Y	Y		
	MCQ170L	Do you still have a liver condition	Y			

	Code	Enrichment name	Raw	Norm	Z-Scal.	AgeAdj
**CLICK Clustering**	DIQ010	Doctor told you have diabetes	Y	Y	Y	Y
	KIQ020	Ever told you had weak/failing kidneys	Y	Y	Y	Y
	MCQ140	Trouble seeing even with glass/contacts		Y	Y	Y
	MCQ160A	Doctor ever said you had arthritis	Y	Y	Y	Y
	MCQ160B	Ever told had congestive heart failure	Y	Y	Y	Y
	MCQ160C	Ever told you had coronary heart disease		Y	Y	Y
	MCQ160D	Ever told you had angina/angina pectoris			Y	
	MCQ160E	Ever told you had heart attack		Y	Y	Y
	MCQ160F	Ever told you had a stroke			Y	
	MCQ160G	Ever told you had emphysema			Y	
	MCQ160L	Ever told you had any liver condition			Y	Y
	MCQ170L	Do you still have a liver condition			Y	Y
	MCQ190	Which type of arthritis = 1 (Rheum. Arth.)			Y	
	MCQ220	Ever told you had cancer or malignancy			Y	
	SMQ040	Do you now smoke cigarettes		Y	Y	Y
	MCQ053	Taking treatment for anemia/past 3 mos				Y

The NHANES code and description of validated terms found in clusters generated by pre-processing with the Z-score normalization method and clustering algorithm with three algorithms (CLICK, K-means and SOM) (top) or using the CLICK clustering algorithm with four pre-processing procedures (bottom). Raw = no transformation; Norm = transformation to normal; Z-score normalization or Z-score normalization with age-adjustment). All the marked terms were enriched significantly (hyper geometric test, P value<0.05) in both the training and validation sets.

### 2.3. The nature of the resulting clusters

**Figure 2:**
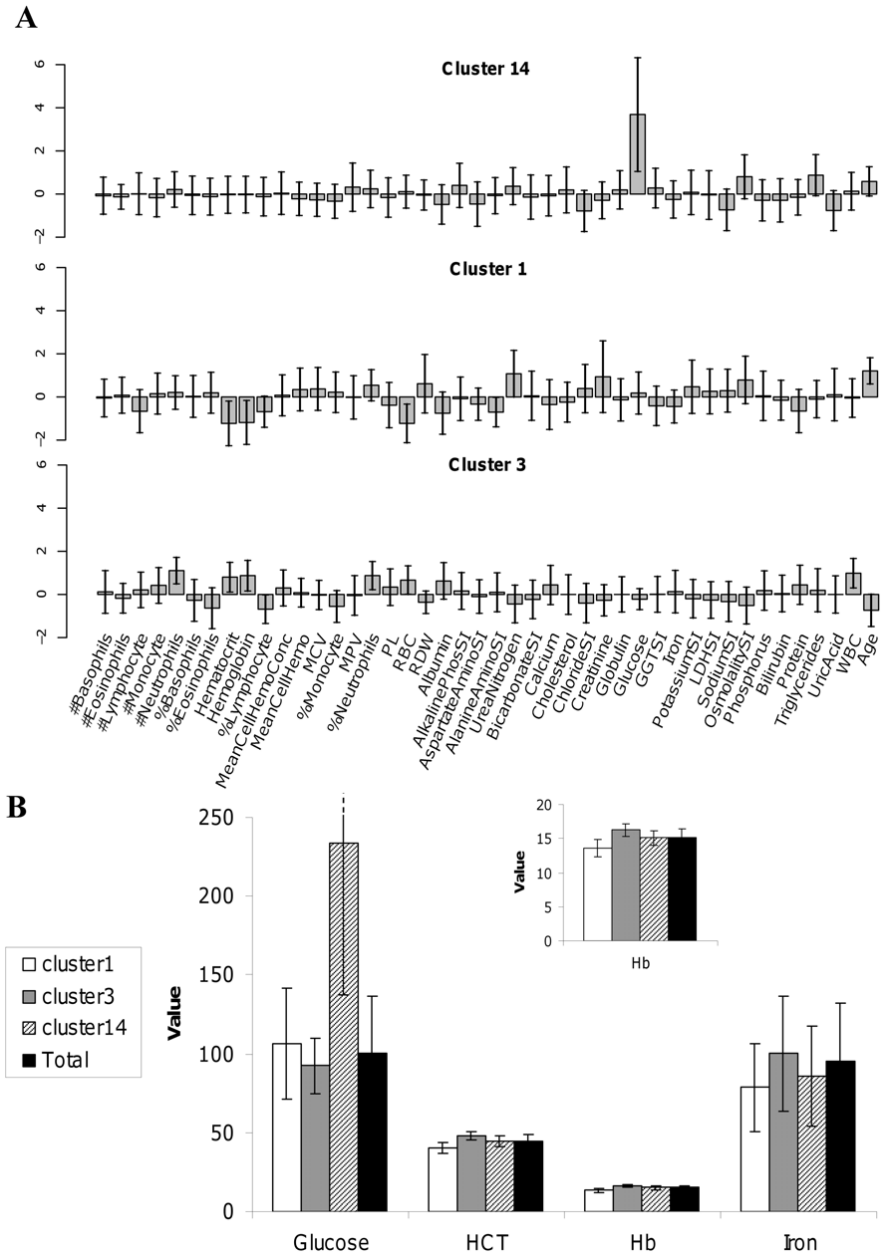
Selected clusters from the NHANES training set. (A) The mean and standard deviation of biomarker values are shown for three selected clusters generated with 4152 males 20 years of age or older from the NHANES training set, using the Z-score normalized/CLICK pre-processing/clustering combination. For each cluster, the total number of individuals (top, right) and selected health/lifestyle traits that are significantly enriched in that cluster (top, left) are provided. For each enriched term, the enrichment factor (i.e. the frequency of the term in a cluster divided by its frequency in the entire dataset) is also provided. (B) Comparison of the original values of selected biomarkers in clusters 1, 3 and 14. The values for Hb are enlarged in the top middle section of the figure.

To test whether the observed clusters can define biomedically homogenous sub-populations, we inspected other cluster characteristics. We hypothesized that if individuals in the same cluster share similar properties, then the enrichment we observed may correspond to a specific pattern of biomarker values in that cluster. To demonstrate that such correspondence can be observed, the mean biomarker values of three clusters were analyzed in the context of their health/lifestyle traits enrichment (Figure 2). Cluster 14 is highly enriched with diabetic individuals. As expected, members of this cluster are characterized by high levels of glucose, blood osmolarity and triglycerides, traits that are indeed hallmarks of unbalanced diabetic patients [7]. Cluster 1 is enriched with individuals suffering from kidney diseases. Cluster members are characterized by a high level of creatinine, increased osmolarity, and low hemoglobin and hematocrit values. While such traits are found in patients with chronic diseases, they are highly suggestive of patients with chronic kidney disease, in particular [8], [9]. Cluster 3 is enriched with smokers, and is characterized by individuals with high hemoglobin, red blood cell and hematorcrit levels, a pattern characteristic of sufferers of lung diseases (e.g., smokers and individuals exposed to severely polluted air [10]). We note that for other clusters, correspondence may be missed if it involves health/lifestyle traits that were not recorded in the NHANES dataset, or if careful inspection by an expert in the appropriate medical field is required to note the correspondence.

### 2.4. Toward novel classification via biomarker-based clustering

Some health/lifestyle states were found to be enriched in multiple clusters (see Table S3). Both clusters 1 and 14, for example, were enriched with diabetic individuals. These clusters, however, greatly differ in terms of characteristic patterns (Figure 2). Mean glucose levels, for example, were very high in cluster 14 but not in cluster 1 (233±95.8 vs. 106±35.4 mg/dL, respectively, p<0.001). This finding, coupled with the iron-related markers, such as hemoglobin, hematorcrit and iron, that were found to be relatively low in cluster 1 but not in cluster 14 (13.6±1.3, 40.4±3.8 and 78.6±28, as compared to 15.1±1.1, 44.8±3.2 and 86.1±32, p<0.001, p<0.001 and p<0.013 respectively) may define a group of better-treated diabetics. A similar pattern is observed for smokers, who are over-represented not only in cluster 3 (as described above) but also in clusters 6, 8 and 12, despite great differences in their respective patterns. It is possible that the stratification of smokers reflects some other conditions or properties of the patients that have a greater impact on biomarker levels. An intriguing possibility is that the unsupervised approach reproducibly clusters sub-populations presenting unique clinical conditions.

**Figure 3:**
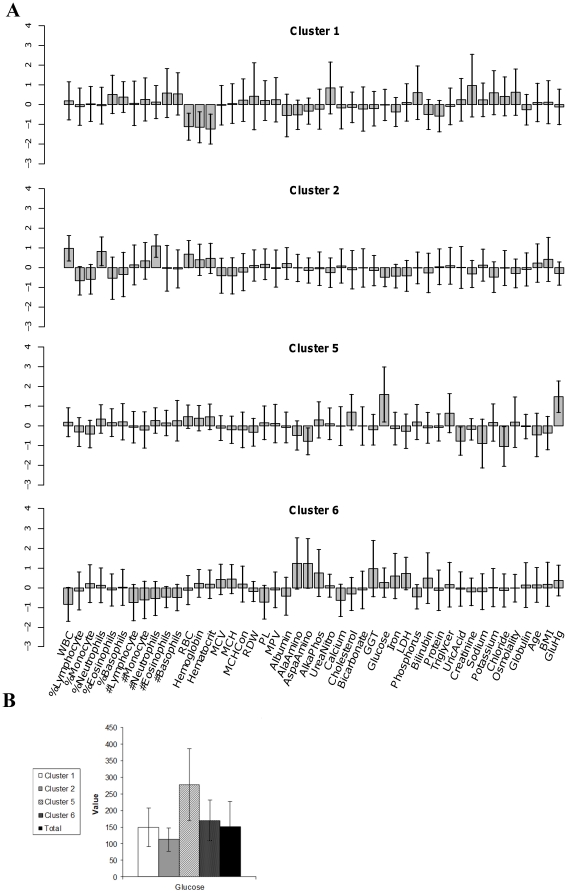
Selected clusters from the NHANES diabetic subset. (A) The mean and standard deviation of biomarker values are shown for four selected clusters generated with 299 males 20 years of age or older from the NHANES training set with self-reported type 2 diabetes mellitus. Clustering was performed using the Z-score normalized with age adjustment/CLICK pre-processing/clustering combination. For each cluster, the total number of individuals (top, right) and selected health/lifestyle traits that are significantly enriched in that cluster (top, left) are provided. For each enriched term, the enrichment factor (i.e. the frequency of the term in a cluster divided by its frequency in the entire dataset) is also provided. (B) Comparison of the glucose levels in clusters 1, 2, 5 and 6.

To further expand and test this hypothesis, we performed unsupervised clustering to detect sub-populations of diabetic individuals. A subset of diabetic individuals (i.e. males, 20 years of age or more) was extracted from the training and the validation sets described above (N = 299 for the training dataset and N = 229 for the validation dataset) by adding two biomarkers that are routinely tested in diabetes patients, namely BMI (body mass index) and glycosylated hemoglobin levels (A1C). The resulting dataset was clustered using the same procedure as described above (Figure 1) but this time using only the CLICK/Z-normalization with age adjustment pipeline. This procedure yielded six clusters involving 245 individuals, with 54 individuals remaining unclassified. To avoid confusion with the clusters discussed above, these clusters are named D_1_ through D_6_. The resulting clusters differ from each other in terms of their mean patterns (Figure 3). Cluster D_2_ members, for instance, are characterized by only slightly elevated glucose levels (with a mean of 111.9±35 mg/dL), cluster D_1_ members are characterized by moderately increased mean glucose levels (mean of 148.9±56 mg/dL), while individuals in cluster D_5_ have very high glucose levels (mean of 277.4±108 mg/dL).

Enrichment analysis relying on health/lifestyle traits described in the NHANES diabetes questionnaire suggests that the observed sub-classification of diabetic patients is indeed meaningful. Cluster D_1_ was found to be enriched with insulin users and with patients suffering from common vascular complications of diabetes, namely, retinopathy (positive answer to the NHANES question “do you have other vision troubles due to diabetes”) and microvascular complications (e.g., “vascular and foot ulcer conditions that required significant time to heal”). In addition, cluster D_1_ is enriched with patients suffering from “weak or failing kidneys”. These enrichments have been successfully reproduced in the validation dataset. In addition to the validated enrichment, an intriguing enrichment was observed for cluster D_6_. This cluster is enriched with individuals that complain of leg pain while walking but not other complaints. The validity of this enrichment could not be assessed, as this complaint was not recorded in those surveys from which the validation dataset was derived.

## Discussion

Unsupervised classification (i.e. clustering) of individuals based on routine blood tests has been previously proposed to allow for the uncovering of patient sub-populations that are seemingly homogenous in terms of clinical and lifestyle traits [5], [6]. In this study, we have demonstrated, for the first time, which clusters resulting from this type of analysis are reproducible across cohorts. Such robustness is essential to allowing clustering to serve as the basis for novel sub-classifying methods. We have demonstrated that a classification scheme developed in one dataset can be used for classifying a new dataset, and that the resulting clusters in the new dataset are most enriched in terms of the same clinical and lifestyle traits.

In addition to exploring the robustness of the clustering approach in clinical applications, we further demonstrated the potential of unsupervised learning to uncover non-trivial sub-classes. As some conditions were found to be enriched in more than one cluster, we believe that a more accurate stratification within a given disease may be achieved with this approach. For example, routine lab tests defined a sub-cluster of diabetic patients enriched with patients complaining of leg pains while walking, a common early indicator of peripheral artery disease (PAD). PAD is a frequent complication of diabetes involving peripheral arterial dysfunction and is associated with an elevated risk of cardiovascular events, amputations, and a general decline in a patient's quality of life. The early diagnosis and treatment of PAD in patients with diabetes may have significant and profound clinical implications [11]. Our results may provide an early indication that PAD can be diagnosed from routine blood tests, although this has yet to be validated with an independent dataset. However, patients suffering from pains are more likely to use analgesics, which, in turn, are known to alter normal values for some clinical biomarkers (e.g., hemoglobin [12]). Moreover, leg pains, as well other muscle pains, may be a side effect of the use of statins [13]. Thus, the clinical value of such results should be best investigated using data from longitudinal studies, after omitting statins. Using the artificial neural network described above, diabetic patients belonging to the same cluster can be identified. If the pattern of blood test values is indeed predictive of PAD, this pattern should precede the use of analgesics or complaints about leg pains. Unfortunately, the datasets we have used throughout this study are cross-sectional, and do not separate the disease from various treatments. Another possible advantage of using longitudinal data is the availability of multiple observations from the same individual. This allows temporal patterns to be considered. Temporal patterns have been successfully used in supervised machine-learning with clinical data [14]. Our results, however, indicate that it may be also useful to study temporal patterns in clinical data using unsupervised learning. Unsupervised learning allows suggesting new stratification schemes, rather than learning how to classify patients according to known strata. We would thus propose that adding temporal analysis into unsupervised learning may help detect new patient stratification schemes.

Our results indicate that age adjustment has an inconsistent effect on clusters quality. On the one hand, clustering the entire population with age-correction decreased the number of clusters without degrading their compactness (i.e. <5% effect on *H_A_*-*S_A_*), yet also decreased the number of distinct valid enrichments by 25% or more. On the other hand, in the diabetic population, age-correction doubled the number of distinct valid enrichments (from 2 to 4) without changing the number of clusters and with little impact on clusters compactness (i.e. <5% change in *H_A_*-*S_A_* values). This suggests that the clusters obtained with age-corrected marker values are more biologically uniform for diabetic patients and less uniform for the general population. These inconsistencies can be explained, at least in part, by complex and non-linear interactions between age and marker levels [15], [16], which can interfere with the relatively simple linear correction method we have used in this study. In fact, we have shown that age effects are more linear in advanced ages (>50), making linear age correction more appropriate in generally older diabetic patients. Age may also have indirect affects on marker levels via its association with various diseases, co-morbidities and non-pathological conditions that, in turn, effect marker levels. Further research is required to better understand how and when age effects need to be corrected for. Toward this goal, we have recently described a database for recording age-disease interactions from biomedical publications [17]. With this resource and others, more refined models for age correction could be developed that will further enhance the power of unsupervised learning in clinical data.

Another aspect of this work is the applicability of Expander, a tool specifically designed for microarray analysis [18], to clinical data analysis. It has been previously suggested that the methodology used to analyze large, multi-dimensional datasets in biological settings (e.g., transcription profiles) is perfectly suited to the processing of clinical biomarkers data [10]. However, the suitability of such tools for this task had not been directly evaluated, and the effect of the parameter choice (e.g., clustering algorithm) was not evaluated. Using a standard microarray analysis tool with only minor adjustments to the pre-processing pipeline, we were able to cluster thousands of individuals, to visualize the results and to test the resulting clusters for enrichment. This means that the arsenal of computational tools available for analysis of biological data, which are characterized by high noise tolerance and performance suitable for large datasets, can be used for medical data analysis.

Studying large databases from medical records using bioinformatics tools carries the hope of revitalizing efforts already in place to implement a more personalized form of medicine. In all likelihood, it will take some time before genetic, proteomics and other novel markers that are being developed for this purpose reach the cutting edge of medical practice. With the findings reported here, we have demonstrated that some of the goals of personalized medicine may have already been met using classical clinical biomarkers. In the past, analysis of these classical markers was limited to the methods of available linear and additive models. Moving toward novel methodologies that make use of non-linear and non-additive models to uncover cryptic sub-populations may help identify groups of patients that are more likely to respond optimally to a more tailored treatment. Bioinformatics tools are most suitable for such analyses, as they incorporate computational analysis methods specifically chosen for their ability to handle noisy, error-ridden and often biased data.

Basic biological research might also benefit from the knowledge generated by patient clustering based on clinical biomarkers. The study of processes such as aging, for example, could benefit from such analysis [15]. As one of the best sources of data for studying inter-individual variation, clinical data offer the possibility to study hundreds of phenotypic biomarkers recorded from many individuals. The clusters we have described here can be used to dissect the sources of variation in clinical markers, pointing to common causes that result in similar differences in multiple individuals.

We believe that further research is required to explore the approach introduced here, to optimize the process for clinical and biological research applications, and to investigate its potential for significant discoveries in clinical and biological research. The results obtained suggest that this novel methodology may provide biomedical researchers with insight not previously available.

## Materials and Methods

### Data sources

Data from the National Health and Nutrition Examination Survey (NHANES), including the 1999–2002 datasets (the ‘training dataset’) and the 2003–2006 datasets (the ‘validation dataset’), were used in this study. Biomarker values were extracted from the relevant files in both sets, omitting markers not routinely tested in clinical settings (e.g., serum vitamin C levels). To avoid the gender differences that occur in many biomarkers and the non-linear and complex changes associated with childhood [15], only males 20 years of age or older were considered. After omitting redundant variables (i.e. the same measurement provided with different units), a matrix with 44 variables (Table S1a) over 4151 or 4225 individuals was extracted for the training and validation datasets, correspondingly.

### Pre-processing

**Figure 4:**
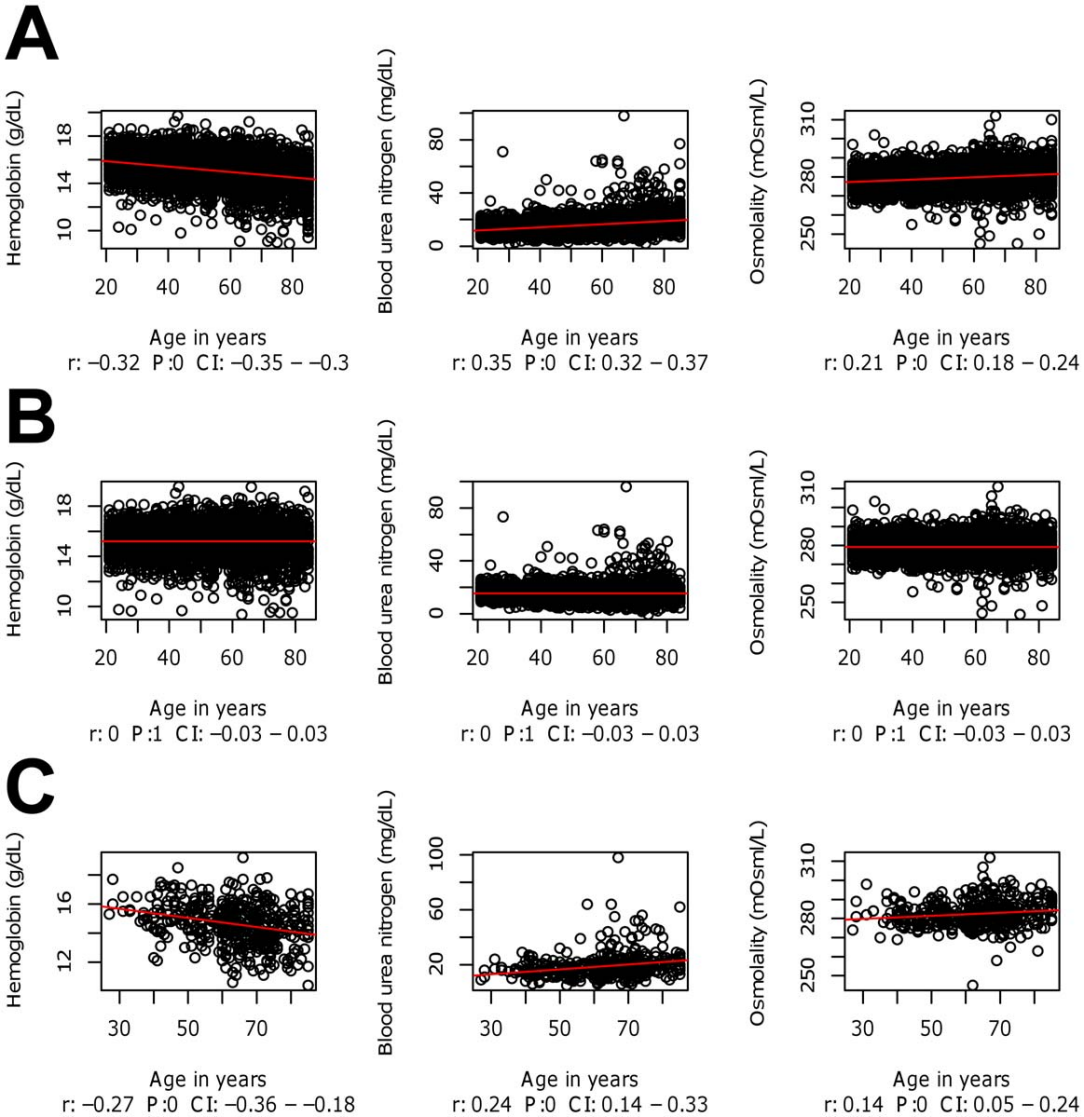
The correlation between selected blood markers and age. Linear regression was calculated for all biomarkers, and least square regression lines (red) were fitted for each marker. r – Pearson correlation coefficient, P – p value, CI- confidence interval of the p-value. (A) Raw data from the training set; (B) training set data after age adjustment. (C) diabetic males, raw data from the training set.

Three derived datasets were generated from the raw data, applying increasingly rigorous pre-processing by aggregating the following procedures: (a) Normal transform of markers with non-normal distribution of values (see Table S1a). Normality was estimated using the Skewness value for each blood marker, Skewness values between −1 to 1 indicated normal distribution [19], (b) scaling each marker to the standard normal distribution, the so-called Z-score transformation, as implemented in R [20], and (c) applying linear-regression-based age correction for those variables found to significantly correlate with age (using the Pearson correlation coefficient, p<0.05 and r>±0.1), as illustrated in Figure 4 and described in full in Table S2.

### Clustering and enrichment analysis

The K-means, SOM and CLICK algorithms, as implemented in Expander (version 4.1), were used for clustering [18]. First, the CLICK algorithm was used since it requires no prior knowledge of the number of expected clusters. To facilitate the comparison between clustering algorithms, the number of clusters detected by CLICK was used as the expected number of clusters for K-means clustering and as the total number of array cells in SOM. To compare the performance of algorithms and pre-processing methods, the mean and SD of the H_A_-S_A_ measure were calculated using a re-sampling approach (removing 3% of the data for each run, N = 10). The statistical significance of differences in H_A_-S_A_ values was estimated using Student's t- test. Enrichment analysis was conducted using the General Enrichment Analysis option of Expander.

### Machine-learning

A neural network model was trained on the cluster assignment of individuals from the training set, and was used to assign individuals from the validation set into clusters. Implementation of the ANN algorithm in the Clementain package (Clementine 10.0; SPSS Inc., Chicago, IL, USA) was conducted, with the *exhaustive prune* option selected.

## Supporting Information

Table S1 **Descriptive statistics.** (**A**) Details of the biomarkers used in this study. Several statistical properties of each biomarker are provided, including the number of observations (N), minimal (min) maximal (max) and mean (mean) values, as well as the standard deviation (Std Dev). ^L^Biomarker is natural-log transformed in the appropriate pre-processing/clustering pipeline; ^A^Biomarker is linearly adjusted for age in the appropriate pre-processing/clustering. (B) The statistical properties of Health/life style variables used for enrichment analysis. For each variable, the code, a short description and the number of subjects that answered positively are recorded for both the training and the validation sets.
(XLS)

Table S2 **Details of the age adjustment process.** For each variable, the correlation coefficient and the parameters used for age adjustment parameters are provided. Age adjustment is performed using the equation *V*′ = *V*−(a*age+*b*)+*m* where *V*′ is the adjusted value, *V* is the raw value and *a*, *b* and *m* are the adjustment parameters.
(XLS)

Table S3 **Complete results of the enrichment analysis.** Details of the enrichment analysis results for all health/life style variables in the training and validation sets. Results are presented with four methods of pre-processing and 3 clustering algorithms. For each analysis pipeline, results are presented for the training set (“1999–2002”) and for the validation set (“2003–2006”). For each trait, information about the cluster (cluster number, size and the number of individuals with the trait that are in the cluster) are provided as well as information about the trait (NHANES code, the actual question that was asked and the total number of individuals who responded positively to the questions in the dataset) and information about the enrichment (p-value and enrichment factor). Enrichment is calculated as the frequency of the term in a cluster divided by its frequency in the entire dataset.
(XLS)
